# Age, Childhood Trauma, Post-Traumatic Stress Disorder, and Substance Use Disorder in the Deep South

**DOI:** 10.1093/geroni/igab046.2021

**Published:** 2021-12-17

**Authors:** Rebecca Allen, Candice Reel

**Affiliations:** 1 University of Alabama, Tuscaloosa, Alabama, United States; 2 The University of Alabama, Tuscaloosa, Alabama, United States

## Abstract

We examined the relationship of reported childhood trauma and PTSD symptoms in a sample (N = 105) of individuals aged 19 to 80 receiving treatment for substance use and opioid use disorders in federally qualified health centers. Fifty-two percent of the sample was age 39 or younger, 30% were in their 40s and 18% were aged 50 and older. Thirty-two percent did not graduate high school and 36% had a high school education or equivalent. Seventy percent reported experiencing adverse childhood experiences (ACES). Although harmful alcohol use was low, 83% of the sample reported substantial or severe substance use, with 41% of the total reporting opioid use. ACES predicted current PTSD symptoms. Telehealth treatment considerations include: 1) internet access, 2) health and mental health literacy, and 3) monitoring for dissociation when using mindfulness-based relapse prevention treatment.

